# *Trichoderma hamatum* and Its Benefits

**DOI:** 10.3390/jof9100994

**Published:** 2023-10-08

**Authors:** Rathna Silviya Lodi, Chune Peng, Xiaodan Dong, Peng Deng, Lizeng Peng

**Affiliations:** Key Laboratory of Agro-Products Processing Technology of Shandong Province, Key Laboratory of Novel Food Resources Processing Ministry of Agriculture, Institute of Food & Nutrition Science and Technology, Shandong Academy of Agricultural Sciences, Jinan 250100, China; silviya.lodi@gmail.com (R.S.L.); pengchune@saas.ac.cn (C.P.); dxd1994@126.com (X.D.); dengpeng2017@163.com (P.D.)

**Keywords:** *Trichoderma hamatum* (Bonord.) Bainier, antimicrobial activity, antioxidant activity, insecticidal activity, herbicidal activity, plant growth promotion

## Abstract

*Trichoderma hamatum* (Bonord.) Bainier (*T. hamatum*) belongs to Hypocreaceae family, *Trichoderma* genus. *Trichoderma* spp. are prominently known for their biocontrol activities and plant growth promotion. Hence, *T. hamatum* also possess several beneficial activities, such as antimicrobial activity, antioxidant activity, insecticidal activity, herbicidal activity, and plant growth promotion; in addition, it holds several other beneficial properties, such as resistance to dichlorodiphenyltrichloroethane (DDT) and degradation of DDT by certain enzymes and production of certain polysaccharide-degrading enzymes. Hence, the current review discusses the beneficial properties of *T. hamatum* and describes the gaps that need to be further considered in future studies, such as *T. hamatum*’s potentiality against human pathogens and, in contrast, its role as an opportunistic human pathogen. Moreover, there is a need for substantial study on its antiviral and antioxidant activities.

## 1. Introduction

*Trichoderma* is a genus of the Hypocreaceae fungal family in which the sexual (telomorphic) stage is referred to as the *Hypocrea* genus and the asexual (anamorphic or mitosporic) stage is referred to as the *Trichoderma* genus [1,2,3]. There are more than 400 species in the *Trichoderma* genus. Some of the prominently distributed species complexes are *Trichoderma harzianum* Rifai, *sensu lato* (THSC) (*Trichoderma harzianum* species complex), *Trichoderma inhamatum* Veerkamp and W. Gams, *T. virens* (J.H. Miller, Giddens, and A.A. Foster), Arx, *Trichoderma spirale* Bissett, *Trichoderma koningii* Oudem, *Trichoderma atroviride* Bissett, *Trichoderma hamatum* (Bonord.) Bainier, *Trichoderma reesei* E.G. Simmons, *Trichoderma viride* Pers., *Trichoderma ghanense* Yoshim. Doi, Y. Abe and Sugiyama, *Trichoderma brevicompactum* G. F. Kraus, C.P. Kubicek and W. Gams, *Trichoderma crassum* Bissett, *Trichoderma erinaceum* Bissett, C.P. Kubicek and Szakcs, *Trichoderma gamsii* Samuels and Druzhinina, *Trichoderma rossicum* Bissett, C.P. Kubicek and Szakacs, *Trichoderma tomentosum* Bissett, *Trichoderma koningiopsis* Samuels, C. Suarez and H.C. Evans, *Trichoderma asperellum* Samules, Lieckfeldt and Nirenberg, and *Trichoderma viridenscens* (A.S. Horn and H.S. Williamson), Jaklitsch and Samuels [4,5]. As the microbial taxonomy rapidly expands, there is a need for fast identification using the available data. Hence, *Trichoderma* spp. has been identified through online by a multilocus identification system (MIST) [6]. Through this method, nearly 349 *Trichoderma* spp. were identified based on the DNA barcodes. Specifically, 44 species of *Trichoderma* were identified based on two genes: RNA polymerase subunit 2 (*rpb2*) and translation elongation factor 1-alpha (*tef1*) [6]. Hence, it is suggested that MIST could be proposed as an appropriate method for obtaining automated species identification through publicly available data [6]. *Trichoderma* spp. diversity distribution has been analyzed in forestry, grasslands, wetlands, and agricultural ecosystems in China. Fifty species have been isolated and identified, among which THSC is the most prominently distributed species. Additionally, there are several other species, including *Hypocrea semiorbis*, *T. epimyces,* Jaklitsch, *T. konilangbra,* Samuels, Petrini, and C.P. Kubicek, *T. piluliferum,* J. Webster and Rifai, *T. pleuroti,* S.H. Yu and M.S. Park, *T. pubescens,* Bissett, *T. strictipilie,* Bissett, *T. hunua* (Dingley), Jaklitsch and Voglmayr, and *T. oblongisporum,* Bissett. The distribution of these species has been well established in northeastern China in Jilin and Heilongjiang provinces, and very few distributions have been observed in Qinghai Province [7]. Studies on *Trichoderma* diversity in aquatic plants and in the soil of Southwest China revealed 23 new *Trichoderma* spp., found by Z. F. Yu, Y. F. Lv, and X. Du: *Trichoderma achlamydosporum*, *Trichoderma amoeum*, *Trichoderma anaharzianum*, *Trichoderma anisohamatum*, *Trichoderma aquatica*, *Trichoderma asiaticum*, *Trichoderma asymmetricum*, *Trichoderma inaequilaterale*, *Trichoderma inconspicuum*, *Trichoderma insigne*, *Trichoderma obovatum*, *Trichoderma paraviride*, *Trichoderma pluripenicillatum*, *Trichoderma propepolypori*, *Trichoderma pseudoasiatium*, *Trichoderma pseudoasperelloides*, *Trichoderma scorpioideum*, *Trichoderma simile*, *Trichoderma subazureum*, *Trichoderma subuliforme*, *Trichoderma supraverticillatum*, *Trichoderma tibetica,* and *Trichoderma unicinatum*. Further studies on these species would be beneficial [8]. 

The biological control of plant pathogens by potential endophytes is increasing. These include *Trichoderma* spp., and they are available in diverse habitats and possess various interactions with other organisms [9]. For example, recent studies on *Codonopsis pilosula,* a Franch Chinese medicinal plant root, shows that endophytes possess antimicrobial activity against human pathogens [10]. *Trichoderma* spp. is highly available in all types of soils, and most of these species are avirulent and opportunistic fungi [11]. *Trichoderma* spp. interacts with other microorganisms, arthropods, and plants in the rhizosphere, causing multi-trophic, interactive networks [12]. *Trichoderma* spp. penetrates into the root systems of plants, survives in the tissues, and is distributed in the aboveground plant parts [9]. Most of these fungi are potential endophytes of several plants and protect plants from various plant pathogens [13].*Trichoderma* spp. directly or indirectly act upon the phytopathogens through several complex mechanisms, such as mycoparasitism, degradation of the pathogen cell wall, competition for nutrients and space, and by inducing resistance in host plants against phytopathogens [14]. *Trichoderma* spp. can be considered as a potential alternative fungicide, reducing the need for synthetic fungicides [15]. However, *Trichoderma* spp., such as *T. longibrachiatum*, *T. viride*, THSC, *T. hamatum*, *T. atroviride*, and *T. koningii*, have been reported to exhibit nematicidal activity [5,16]. *Trichoderma* spp. were considered as the treasure house of several medically important secondary metabolites [17]. *Trichoderma* spp. produces peculiar secondary metabolites, such as peptaibols, which cause pores to emerge in bilayer lipid membranes and, hence, exhibit antimicrobial activity [18]. *Trichoderma* spp. live around and within plants and cause significant alterations in metabolism and changes in certain elements, such as water content, transpiration, and photosynthetic rate; moreover, their presence alters hormone, phenolic compound, amino acid, and soluble sugar contents [19,20]. *Trichoderma* spp. produces several secondary metabolites, a few of which possess significant beneficial effects, including epipoly-thiodioxopiperazines, xylanases, peptaibols, pyrones, polyketides, volatile and nonvolatile terpenes, cerato-plantanins, and siderophores, which are released into the rhizosphere. This leads to enhanced plant growth, stimulating an increase in systemic resistance in plants, and hence surpassing plant pathogens in biocontrol activity [21,22,23]. However, among secondary metabolites, terpenoids possess prominent pharmacological activity with structural diversity; in total, 253 terpenoids have been identified among *Trichoderma* spp. from 1948 to 2022 [24]. *Trichoderma* spp. presented a stable and higher growth rate in soils that were weakly alkaline. *Trichoderma* spp. evinced higher diversity in connection with higher potassium and phosphorous, which indicates that these are prominent edaphic factors for the higher diversity of *Trichoderma* spp. [25]. *Trichoderma* spp. possess biocontrol activity by producing certain antibiotics and hydrolytic enzymes, such as chitinase and β-1,3-glucanase, that facilitate cell wall degeneration and hence cause the death of pathogenic microorganisms [26,27,28]. *Trichoderma* spp. is an efficient fungal species with various benefits and is most prominently researched for its efficacy as a biological fungicide, and it can be used as a potential biocontrol strains [29]. There are several myths and dogmas over biocontrol changes with respect to *Trichoderma* spp. because the biocontrol efficacy depends on several genes and their specificity, hence there is a need to investigate and produce broad-spectrum bioactive agents that are economically friendly and which would be beneficiary to users and buyers [30], whereas *Trichoderma* spp. interacts with *Arabidopsis* plant through root sensing. Rhizosphere acidification by *Trichoderma* spp. triggers root developmental response to auxins, volatile organic compounds (VOCs), and other bioactive molecules; hence, it leads to crop improvement by primary root growth and lateral root formation [31]. 

Moreover, *Trichoderma* spp. are potential decomposers and are significant secondary biofuel producers from cellulosic waste [32,33]. Moreover, *Trichoderma* spp. has been accommodated in the “attine ant environment” as a mutualistic fungal partner. Approximately 20 different *Trichoderma* spp. have been isolated from this environment [34]. However, *T*. *hamatum* had a positive association with *Tricholoma matsutake* (S.Ito and Imai) Singer (pine mushroom) in fairy rings and had higher enzyme activity. This association was due to the degradation of wood litter by these enzymes and provides a carbon source to *Tricholoma matsutake* [35]. The new *Trichoderma* sp. *Trichoderma songyi* (M.S. Park, S.-Y.Oh, and Y.W. Lim) was isolated from pine mushrooms, but its interaction with *Tricholoma matsutake* needs to be studied [36]. 

*Trichoderma* spp. possesses tolerance toward heavy metals such as nickel and cadmium [37]. *Trichoderma* spp. are also used in the production of myco-nanoparticles that can be used as nanofertilizers, nanofungicides, plant growth stimulators, and nanocoatings [38]. Traditional nanoparticle synthesis is expensive and causes the release of hazardous chemicals into the environment [39]. Hence, microbial nanotechnology produces hazard-free nanoparticles that are environmentally friendly.

However, THSC infects *Diaforobiotus* tardigrades, other eutardigrade in the genus *Milnesium*, and heterotardigrades in the genus *Viridiscus* [40]. However, apart from the benefits, a detrimental role of *Trichoderma* spp. was found against edible mushrooms in causing green mold disease, although treatment with antifungal agents such as prochloraz and metrafenone reduced green mold disease on edible mushrooms [41], whereas *Trichoderma* spp. *T. atroviride*, *T. viride*, *T. koningiopsis,* and *T. hamatum* cause adverse effects for moss plants, i.e., *Physcomitrella patens* (Hedw.), Bruch and Schimp, by damaging the protonema, stem, and leaves [42]. 

*T. hamatum* is also prominent *Trichoderma* spp. like other species with various beneficiary properties, whereas some *Trichoderma* spp., such as THSC, *T. viride*, *T. brevicompactum*, etc., can be thought of as opportunistic human pathogens; they cause several opportunistic infections, such as rhino sinusitis, liver infections, pneumonitis, endocarditis, hematological malignancies, hypersensitivity, acute sinusitis, etc. [43]. However, research on *T. hamatum* regarding its opportunistic pathogenicity has not been reported on to our knowledge. This review gives a broad overview of the *T. hamatum* species that possess various beneficial qualities, such as biocontrol activities, plant growth promotion, and several other beneficial activities, and provides a research path for the gaps that need to be studied. Further, research on *T. hamatum* would definitely provide a natural biocontrol and plant growth product that is economically friendly and will be valuable for agricultural uses. 

## 2. Literature Search for *T. hamatum* and Its Analysis

**Figure 1:**
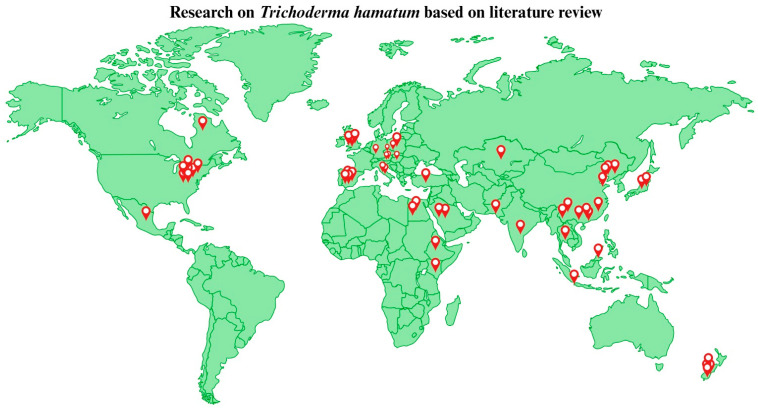
Global distribution of research on *Trichoderma hamatum*.

**Figure 2:**
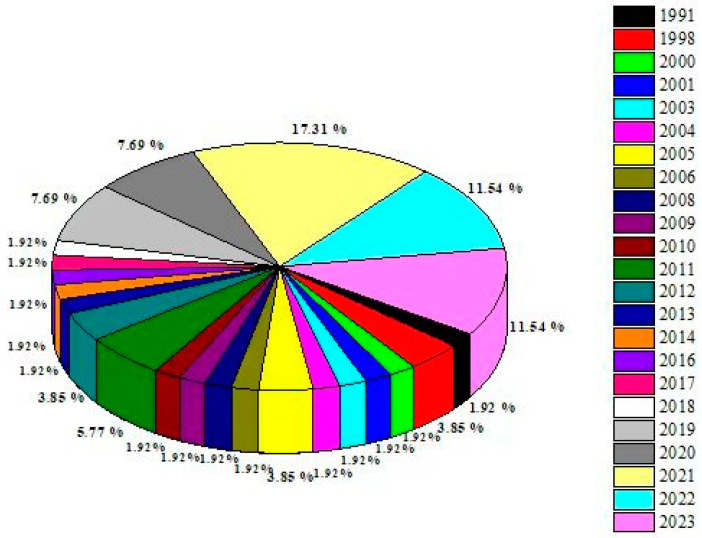
Number of research publications on *Trichoderma hamatum* based on year of publication.

The literature was reviewed for studies published up to 2023 by using the keyword “*T. hamatum*” on Pubmed, Web of Science, cross ref, Elsevier, Springer Link, Google Scholar, and Scopus. The retrieved articles were characterized based on the beneficiary activities of *T. hamatum*, such as the antibacterial, antifungal, antiviral, herbicidal, insecticidal/pesticidal, antioxidant, and plant growth promotion activities, among other beneficiary activities. The content of the review has been arranged and presented in sections. However, with tremendous effort, we have demonstrated almost all of the related research in this review, with some exceptions. Moreover, based on the literature, clear information of the research year and location for each study has been presented (Figure 1 and Figure 2). 

The research we have presented in the current review on *Trichoderma hamatum* was published by authors throughout the world: Austria—2; Canada—1; China—10; Czech republic—1; Egypt—2; Ethiopia—1; Germany—1; Hungary—1; India—1; Indonesia—1; Italy—2; Japan—1; Kazakhstan—1; Kenya—1; Malaysia—1; Mexico—1; New Zealand—4; Pakistan—1; Poland—2; Saudi Arabia—2; Spain—4; Thailand—1; Turkey—1; United States of America—7; United Kingdom—3. 

Research articles represented in the review article have been arranged based on the year of publication in Figure 2. The highest percentages of articles from the current review on *Trichoderma hamatum* were published in 2021, accounting for 17.31% of the total sample. 

## 3. *Trichoderma hamatum*

*T. hamatum* is the fungal species belongs to Hypocreaceae family; its binomial name is *Trichoderma hamatum* (Bonord.), Bainier, 1906. It is a saprophytic fungi and is commonly found in humus, litter, soil, and plant rhizosphere [44]. Morphology of *T. hamatum*: the stroma surface is yellowish-brown or dull orange; the entostroma is white–light brownish; the spores are white. The culture of *T. hamatum* on Castenholz medium D (CMD) and synthetic nutrient pore agar (SNA) lacks pigmentation and odor, and it diffuses on the medium in concentric zones. After 4–5 days of incubation, growth is plentiful, nearly globose, with compact pustules which are 0.4–2 mm diameter. The aggregation turns pale green and the arising pustules are 11 µm wide with 5–6 µm wide branches and 6–7 µm thickness. The conidiophores radiate from the reticulum; the pachybasium at the base is 50–200 µm long and 2–4 µm wide. These are persistent, smooth, thin-walled, straight sinuous, or helically twisted, with slightly pointed elongations. Conidia are 4.0–4.7 × 2.7–30 µm in an oblong shape or they are ellipsoid with parallel sides; these are green, smooth, and have indistinct scars. The asexual morphology is typically of the pachybasium type of conidiophores, as identified by broad branches with ampulliform phialides and frequent occurrence of well-differentiated sterile or fertile elongations of conidiophores, whereas the sexual morphology is similar to other *Trichoderma* spp. The above morphological description of *T. hamatum* is based on previous studies [45,46,47,48,49]. 

## 4. *T. hamatum* and Its Biocontrol Activities

### 4.1. Antibacterial and Antifungal Activity

*T. hamatum* is an endophytic fungus that possesses biological control abilities against several plant pathogens. Bacterial leaf spot of radish, caused by *Xanthomonas campestris* pv. *armoraciae* McCulloch, Dye, was suppressed when the plants were grown in the sphagnum peat mix that possessed strain T_382_ (Table 1). Strain T_382_ induces systemic resistance in the growth of pathogenic bacteria; hence, the disease is controlled [50]. The biocontrol activity of strain T_382_ has been consistently demonstrated in tomato plants by its significant action against *Xanthomonas euvesicatoria* Jones, emnd, Constantin 110c, which causes bacterial spot of tomato. Strain T_382_ induces resistance in tomato plants by modulating gene expression in tomato leaves. The expressed genes were responsible for functions such as biotic and abiotic stress responses and RNA, DNA, and protein metabolism. Hence, due to these gene modifications, systemic resistance was attained in the plants; thus, suppression of disease occurred [51]. *T. hamatum* SU136 culture filtrate, when exposed to different concentrations of gold chloride (0.25, 0.5, and 1.0 mM), formed stable AuNPs; among them, the smallest AuNPs were obtained with 0.5 mM gold chloride. These AuNPs possess antimicrobial activity against four bacterial pathogens: *Bacillus subtilis* ACCB 133, *Staphylococcus aureus* ACCB 136, *Pseudomonas aeruginosa* ACCB 156, and *Serratia* sp. ACCB178 [52]. *T. hamatum* FB10 exhibits antibacterial activity against *Acidovorax avenae* Schaad et al. and *X. campestris,* and antifungal activity against *S. sclerotiorum*, *Rhizoctonia solani*, *Alternaria radicina* Meier, Drechsler and E.D. Eddy, *Alternaria citri,* Ellis and N. Pierce, and *Alternaria dauci* (J.G. Kuhn), J.W. Groves and Skolko, by producing volatile secondary metabolites [53]. Moreover, *T. hamatum* evinced antibacterial activity against bacterial wilt caused by *Ralstonia solanacearum* in *Solanum lycopersicum* L. (tomato) plants. This was represented by analyzing crop mortality rate, incidence, and the area under the disease progression curve [54]. However, the growth inhibition of various bacteria and phytoplanktons were caused by cyclonerane sesquiterpens, such as 5-hydroxyl epicyclonerodiol oxide and 4-hydroxyl epicyclonerodiol oxide, and one naturally occurring halogenated trichothecane derivative, known as trichodermol chlorohydrin, isolated from *T. hamatum* Z36-7, that was obtained from marine red alga *Grateloupia* sp. [55]. 

*T. hamatum* antagonistic fungi follow several mechanisms, such as mycoparasitism, antibiosis, and competition. Damping-off of radish seedlings by *Rhizoctonia solani,* J.G. Kuhn, was controlled by using potting mix that contained *Chryseobacterium gleum* (Holmes et al.) Vandamme et al. (C_299_R_2_) and *T. hamatum* (T_382_) as the biocontrol agents with pine bark mix. However, in other potting mixes, i.e., light and dark peat mixes, the strain C_299_R_2_ number significantly decreased compared to strain T_382_. Hence, the strain T_382_ population contributed to the control of *Rhizoctonia* crown and root rot in poinsettia [56]. Mycoparasitic interactions were studied between the biocontrol agent *T. hamatum* and the phytopathogen *Sclerotinia sclerotiorum* (Lib.), de Bary. These studies revealed that 19 novel genes of *T. hamatum* presented increased expression during mycoparasitism compared to the control. The proteins produced by these genes included three monooxygenases, a metalloendopeptidase, and a glucose dehydrogenase, which are responsible for antifungal activity [57]. *T. hamatum* exhibits antagonistic activity against plant pathogens such as *S. sclerotiorum, Sclerotinia minor,* and *Sclerotium cepivorum* (Table 1)*. T. hamatum* expresses monooxygenase genes in response to these plant pathogens, and disruption of monooxygenase-expressing genes does not affect *T. hamatum* growth, but antagonistic activity is inhibited [58]. However, *T. hamatum* GD12 can perform both biocontrol activity against *S. sclerotiorum* and lettuce plant growth promotion activity at the same time. This biphasic response was analyzed by identifying significantly expressed genes involved in secreting cysteine-rich proteins and secondary metabolites [59]. Moreover, strain GD12 promotes growth in the model dicot *Arabidopsis thaliana* (L.), Heynh, and enhances foliar resistance against the pathogen *Magnaporthe oryzae,* B.C. Couch, which infects monocot rice. Strain GD12 possesses unique genome sequences compared to other *Trichoderma* genomes, which indicates that starin GD12 possesses the potential to encode various novel bioactive compounds. Further analysis of these bioactive compounds would definitely benefit agriculture, if it were possible to enable strain GD12 utilization as a biocontrol agent [60]. 

Moreover, *T. hamatum* and *T. koningiopsis* isolated from Asarum rhizosphere evinced antifungal activity against *Sclerotinia asari* Y.Wu and C.R. Wang. Further, it is proved that non-volatile compounds, such as Abamectin, Eplerenone, Bhenic acid, Josamycin, Erythromycin, Methyleugenol, and Minocycline, evinced significant antifungal activity. Hence, *T. koningiopsis* and *T. hamatum* suggested as biocontrol agents for the treatment of *Asarum sclerotiorum* [61]. However, *Trichoderma* spp. also possesses a disadvantage: the antagonistic property of *Trichoderma* spp. affects not only plant pathogens but also plant-beneficial fungi such as the mycorrhiza-forming species *Laccaria bicolor* (Maire), P.D. Orton, which is a common ectomycorrhizal fungus. *T. hamatum* exhibits antagonistic properties against plant-beneficial fungi by releasing a range of (VOCs) [62]. Root rot caused by fungal pathogens such as *Fusarium proliferatum* (Matsush.), Nirenberg ex Gerlach and Nirenberg, *Fusarium solani* (Mart.) Sacc., and *Fusarium oxysporum,* Schlecht. Emend., Snydere, and Hansen, in *Aconitum carmichaelii,* Debeaux, was controlled by the antagonistic activity of *T. asperellum*, *T. hamatum,* and *Trichoderma virens,* J.H. Miller, Giddens, and A.A. Foster (Table 1). The volatile secondary metabolites produced by these *Trichoderma* spp. possess antifungal activity and hence control root rot disease [63]. The endophytic fungus *T. hamatum* C9 of *Macadamia integrifolia,* Maiden and Betche, revealed antifungal activity against the pathogenic fungus *Lasiodiplodia theobromae* (Pat.), Griffon and Maubl. Its antifungal activity has been proven both in vitro and in vivo [64]. *T. hamatum* was also represented as an interplant communicator; in vivo analysis of the plant *A. thaliana* proved the communication effect of *T. hamatum*. When an *A. thaliana* leaf was infected by *S. sclerotiorum* and *X. campestris*, the jasmonic acid (JA) levels increased in the leaf, which initiated an increase in salicylic acid (SA) levels in the roots; hence, *T. hamatum* colonization was reduced in the plant root. However, in leaf-infected plants, *T. hamatum* communicates from the root of the infected plant to other nearby plants and stimulates an increase in SA in their roots, whereas this increase in SA stimulates an increase in JA levels in the leaves of the nearby plants. Thus, *T. hamatum* naturally increases the plant systemic defense mechanism. Through this mechanism, immunity against foliar infecting pathogens is attained [65]. 

However, downy mildew disease in pearl millet caused by *Sclerospora graminicola* sacc., J. Schrot, has been suppressed by its endophytic fungus *T. hamatum* UoM13. In vitro studies revealed that pearl millet seeds treated with *T. hamatum* UoM13 exhibited significantly increased activity of defense enzymes such as glucanase, peroxidase, phenylalanine, ammonia-lyase, and polyphenol oxidase. Moreover, enhanced expression of endogenous salicylic acid led to systemic immunity in plants through the salicylic acid synthetic pathway [66]. On the other hand, when cucumber transplants were grown in compost-amended potting mix inoculated with strain T_382_, the growth of *Phytophthora capsica,* Leonian, which causes Phytophthora root and crown rot, was also suppressed. Strain T_382_ significantly suppressed Phytophthora leaf blight, similar to benzothiadiazole or mefenoxam [67]. *T. hamatum* K01 evinced antifungal activity against *Colletotrichum gloeosporioides,* (Penz.) Penz. and Sacc., causing anthracnose of citrus by producing certain secondary metabolites, such as pyrone, organic compounds, fatty acids, and sorbicillin [68]. 

### 4.2. Antiviral Activity

*T. hamatum* Th23 endophytes of *S. lycopersicum* (tomato) roots possess antiviral activity against tomato mosaic virus (TMV) (Table 1). Soil pretreatment with *T. hamatum* Th23 before TMV inoculation evinced a reduction in TMV accumulation in the plant. *T. hamatum* Th23 significantly increased the activity of protective scavenging enzymes such as polyphenol oxidase (PPO), heme-containing catalase (CAT), and superoxide dismutase (SOD) and simultaneously decreased the levels of nonenzymatic stress markers such as hydrogen peroxide (H_2_O_2_) and malondialdehyde (MDA). Moreover, systemic resistance was enhanced in plants treated with *T. hamatum* Th23 through increases in the transcription of polyphenol genes such as hydroxycinnamoyl CoA quinate transferase (HQT) and chalcone synthase (CHS) and pathogen-related genes such as pathogen-related proteins 1 and 7 (PR-1 and PR-7). *T. hamatum* Th23 also induced tomato plant growth by increasing shoot and root parameters and chlorophyll content in the plant [69].

### 4.3. Insecticidal or Pesticidal Activity

*T. hamatum* evinced its antagonistic insecticidal activity against one of the most common pests, commonly called cotton leaf worm, or scientifically called *Spodoptera littoralis* Boisduval, which causes enormous commercial losses to horticultural and ornamental crops in the greenhouse setting. The spores and culture filtrate of *T. hamatum*, when ingested by the larvae of *S. littoralis*, caused high mortality. However, the antagonistic activity was mainly due to the production of the siderophore rhizoferrin by *T. hamatum*, as proven by metabolomics analysis of the culture filtrate [70]. *T. hamatum* FB10 synthesizes secondary metabolites that possess nematicidal activity against *Meloidogyne incognita* Kofoid and White by inhibiting egg hatching at 78 ± 26% and promoting mortality at the juvenile stage at 89 ± 2.5% [53] (Table 1). However, *Trichoderma* spp. such as *Trichoderma longibrachiatum,* Rifai, *T. koningii*, *T. hamatum*, *T. atroviride*, *Trichoderma spirale,* Indira and Kamala, THSC, and *T. viride* protect termites (*Coptotermes formosanus* Shiraki) from the entomopathogenic fungus *Metarhizium anisopliae* (Merschn), Sorokin. Analysis of the termite’s aggregation and tunneling behavior with soil/sand treated with conidia of this *Trichoderma* spp. demonstrated that soil/sand treated with conidia of *Trichoderma* spp. aggregated more termites compared to untreated soil/sand [71]. *Trichoderma* spp. *T. longibrachiatum, T. koningii, T. hamatum, T. atroviride, T. viride,* and *T. spirale* significantly reduced the tunneling of *Odontotermes formosanus* (Shiraki) in the sand (Table 1). However, choice test evinced that *T. koningii*, *T. atroviride,* and *T. spirale* repelled *O. formosanus* aggregation, but *T. longibrachiatum* and *T. hamatum* attracted termites. Hence, it is suggested that soil or root of seedlings pretreated with *Trichoderma* spp. could protect the plants from *O. formosanus* infestation [72]. Entomopathogenic *Trichoderma* spp. THSC, *T. hamatum,* and *T. asperellum* exhibited pesticidal activity against *Ceratovacuna lanigera,* Zehnter (Hemiptera: Alphidiae), which destructs sugarcane (Table 1). However, THSC evinced 75.70% and 72.31% mortality rate of nymph and adult pest, respectively; *T. hamatum* contributed 63.56% and 60.91% [73], respectively; *Trichoderma* spp. THSC, *T. hamatum*, *T. asperellum,* and *T. atroviride* evinced antifungal activity on symbiotic fungus of *Xylosandrus germanus* Blandford causing a decrease in brood production and the simultaneous suppression of the insect population [74] (Table 1). 

**Table 1:** *Trichoderma hamatum* and its biocontrol activities.

Activity	Host/Source	Pathogen	Reference
Antibacterial	Radish	*Xanthomonas campestris* pv. *armoraciae*	[50]
	*S. lycopersicum*	*Xanthomonas euvesicateria*	[51]
	n.a	*Bacillus subtilis* *Staphylococcus aureus* *Pseudomonas aeruginosa* *Serratia*	[52]
	n.a	*Acidovorax avenae* *Xanthomonas campestris*	[53]
	*S. lycopersicum*	*Ralstonia solanecearum*	[54]
	*Grateloupia* sp.	Phytoplantons and several bacteria	[55]
Antifungal activity	Radish	*Rhizoctonia solani*	[56]
		*Sclerotinia sclerotiorum* *Sclerotinia minor* *Sclerotinia cepivorum*	[57,58]
	Lettuce	*Sclerotinia sclerotiorum*	[59]
	*Arabidopsis thaliana*	*Magnaporthe oryzae*	[60]
	*Asarum* Rhizosphere	*Sclerotinia asari*	[61]
	n.a	*Laccari bicolor* (Mycorrhiza forming species)	[62]
	*Aconitum carmichaelii* Debx	*Fusarium proliferatum* *Fusarium solani* *Fusarium oxysporum*	[63]
	*Macadamia integrifolia*	*Lasiodiplodia theobromae*	[64]
	*Arabidopsis thaliana*	*Sclerotinia sclerotiorum*	[65]
	Citrus	*Colletotrichum gloeosporiodies*	[68]
Antioomycete	Peal millet	*Sclerospora germinicola*	[66]
	Cucumber	*Phytophthora capsici*	[67]
Antiviral	*S. lycopersicum*	Tomato mosaic virus	[69]
Insecticidal/Pesticidal activity	Horticultural and ornamental plants	*Spodoptera littoralis*	[70]
	n.a	*Meloiogyne incognita*	[53]
	n.a	*Odontotermes formosanus*	[72]
	Sugar cane	*Ceratovacuna lanigera*	[73]
	n.a	*Xylosandrus germanus*	[74]
Herbicidal	n.a	*Bidens pilosa*	[75]

*T. hamatum* is a prominent fungus that exhibits several biocontrol activities, including antifungal, antibacterial, antiviral, insecticidal, and herbicidal activities. The table describes the hosts of *T. hamatum* and the susceptible pathogens. “n.a” refers not available.

### 4.4. Herbicidal Activity

Synthetic herbicides have negative impacts on farming and have several side effects. Hence, these synthetic herbicides need to be replaced by biological herbicides using several useful biocontrol fungi. One study revealed that conidial suspensions of *Aspergillus niger* van Tieghem, *T. asperellum*, *T. atroviride*, *T. hamatum*, THSC, and *T. viride* possess herbicidal activity. These conidia affect seed germination and the early growth of the target weed *Bidens pilosa* L.1753 when compared to untreated plants [75] (Table 1). *T. inhamatum* can potentially be used as a biological decomposer of glyphosate in soils where large amounts of glyphosate-containing nonselective herbicides have been used [76].

## 5. Antioxidant Activity

Antioxidants are compounds that are available in plants, and these antioxidants neutralize harmful free radicals in the human body. However, *T. hamatum*, when applied to the roots of *Brassica* crops such as kale, cabbage, leaf rape, and turnip greens, there was increase in aliphatic glucosinolates, such as glucoiberin and sinigrin and indole glucobrassicin in cabbage and gluconapins increased in turnip greens, whereas antioxidant phenolic compounds significantly increased in the leaves of cabbage and turnip greens after root inoculation [77].

## 6. *T. hamatum* and Its Up-Land Plant Growth Promotion Capability

**Figure 3:**
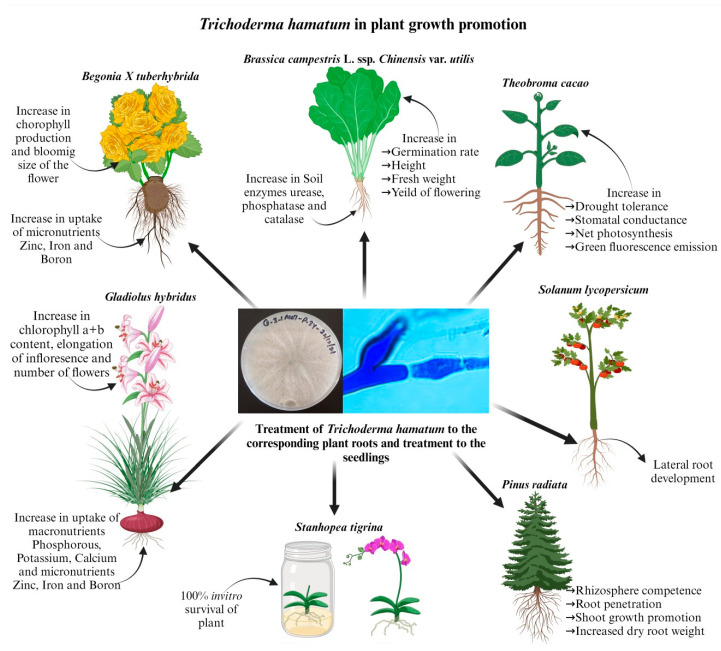
Upland plant growth promotion activity of *Trichoderma hamatum*.

*T. hamatum* DIS 219b affected *Theobroma cacao* L. (cacao) exposed to drought stress, and the plants that were colonized by *T. hamatum* DIS 219b had more tolerance toward drought conditions through alterations in stomatal conductance, net photosynthesis and green fluorescence emissions (Figure 3 and Table 2). All these changes were observed by studying the altered expression of 19 expressed sequence tags (ESTs) that were analyzed by reverse-transcription PCR. However, EST expression in roots has less influence on EST expression than that in leaves. Nine-day-old seedlings colonized by *T. hamatum* DIS 219b had significant expression of the ESTs in the root and increased root fresh and dry weight and root water content. Moreover, the contents of the amino acids such as alanine and γ-aminobutyric acid increased in the leaves, with simultaneously decreased aspartic acid and glutamic acid contents. Hence, the colonized seedlings presented delayed wilting and drought resistance, and root growth promotion was triggered [78]. *Pinus radiata* D. Don seedlings were treated with *T. hamatum* LU592 either by seed coat or spray application to induce rhizosphere competence and root penetration (Figure 3 and Table 2). However, the treated seedlings showed significantly reduced mortality, by up to 29%, shoot growth was promoted by 16%, and height and root dry weight were increased up to 31% compared to application of another *Trichoderma* sp., i.e., *T. atroviride* LU132 [79]. However, in addition to the benefits of *Trichoderma* spp., there are also disadvantages. *S. lycopersicum* plants treated with several *Trichoderma* spp., THSC (T34), *T. virens* Gv29-8 (T87), and *T. hamatum* MI224801 (T7) affected the growth of lateral roots in *S. lycopersicum* plants in various ways, which was proven by in vitro and in vivo assays. Strain T7 and strain T34 have beneficial effects on *S. lycopersicum* seedlings and lateral root development, but strain T87 has detrimental effects on the growth of *S. lycopersicum* seedlings and lateral root development [80] (Figure 3 and Table 2). *T. hamatum* LU592 was transformed with green fluorescent protein (gfp) and hygromycin B resistance genes to monitor the plant health and growth of *P. radiata*. *T. hamatum* LU592 colonized the roots of the plant with 10^3^, 10^5^, and 10^7^ spores per pot. Interestingly, 10^5^ spores per pot yielded effective colonization of the rhizosphere compared to the other concentrations. There was a positive relation between *T. hamatum* LU592 and root maturation with 10^5^ spores per pot inoculum, which correlates with the spatial and temporal proliferation of *T. hamatum* LU592 in the root system of *P. radiata* [81]. 

Moreover, the four *Trichoderma* spp., i.e., THSC, *T. asperellum*, *T. hamatum*, and *T. atroviride* were together used as biofertilizer for 30 days to *Brassica campestris* L. ssp. *chinensis* var. *utilis,* Tsen et Lee (flowering Chinese cabbage) (Figure 3 and Table 2). There was significant increase in germination rate, height, fresh weight, and yield of flowering Chinese cabbage compared to the control. Moreover, there was also significant increase in soil enzymes on the 30th day, such as urease, phosphatase, and catalase [82]. However, *Trichoderma* spp. *T. viride*, THSC, and *T. hamatum* together when treated with the tubes of *Begonia X tuberhybrida* Voss. ‘Picotee Sunburst’ before planting resulted in enhanced blooming size of the flower (Figure 3 and Table 2). Moreover, they stimulated the production of chlorophyll and uptake of microelements, such as zinc, iron, and boron [83]. Moreover, suspension of *Trichoderma* spp., i.e., *T. viride*, THSC, and *T. hamatum,* applied on 5-week-old cultivation of *Gladiolus hybridus,* ‘Advances Red’, resulted in improved macronutrients (P, K, and Ca) and micronutrients (Zn, Fe, and B) uptake (Figure 3 and Table 2). Moreover, there was increase in chlorophyll a + b content in leaves and increased elongation of inflorescence shoot and inflorescence, thus resulting in a higher number of flowers [84]. The endangered species of orchid *Stanhopea tigrina* evinced 100% survival on treatment of vitro plants with *T. hamatum* spore suspension (Figure 3 and Table 2). The symbiotic association of *T. hamatum* was effective in in vitro propagation of *S. tigrina* [85]. 

Treatment of *Trichoderma hamatum* to the roots or seedlings of different plants, such as *Brassica campestris* L. spp., Chinensis var. utilis, Theobroma cacao, *Solanum lycopersicum*, *Pinus radiata*, *Stanhopea tigrina*, *Gladiolus hybridus,* and Begonia *X tuberhybrida* causes plant growth promotion and several other beneficial effects.

## 7. Other Benefits of *T. hamatum*

*T. hamatum* produces the secondary metabolite 4,6-dihydroxy-5-methoxy-6a-methylcyclohexa [*de*] indano-[7,6-*e*] cyclopenta [c] *2H*-pyran-1,9-dione, which inhibits the enzyme 5’-hydroxyaverantin dehydrogenase essential for aflatoxin biosynthesis [86] (Table 3). *T. hamatum* secretes polysaccharide-degrading enzymes such as the hemicellulose enzyme α galactosidase (AGL) and the cellulase enzyme endo-1,4 β glucanase (EG) (Table 3). These enzymes were quantified using monoclonal antibodies, and the enzyme activity assays and enzyme protein concentrations were analyzed by enzyme-linked immunosorbent assay (ELISA). Hence, using these secretions, *T. hamatum* competes for nutrients in natural environments, increasing their growth and suppressing the growth of plant pathogens [87]. *T. hamatum* NGL1 produces endoglucanase by using cow dung in solid state fermentation. The endoglucanase produced *T. hamatum* NGL1 evinced saccharification efficiency as commercial enzyme [88] (Table 3). *T. hamatum* possesses chitinase activity, but when the *T. hamatum* strain Tam-61 was transformed with the 42-kDa endo chitinase-encoding gene *Tam-ch*, the transformed fungus presented higher chitinase activity than the wild type (Table 3). This indicates that biocontrol capability can be enhanced by using transformed *T. hamatum* [89]. *Trichoderma* spp. possesses cellulose-degrading capacity, but the potential of β-glucosidases differs in different species and strains. *T. hamatum* YYH13 and YYH16 have been shown to differ in cellulose degradation efficiencies. Hence, when their genomes were analyzed, 15 protease genes differed between YYH13 and YYH16. YYH13 possesses 10 families of carbohydrate-active enzymes along with the GH1, GH3, GH18, GH35, and GH55 families of chitinase, glucosidase, galactosidases, and glucanase. Hence, YYH13 has greater cellobiose-hydrolyzing efficiencies compared to YYH16. This result indicates that every strain of *T. hamatum* possesses unique activity potentials (Table 3). Moreover, β-glucosidase gene expression was higher in YYH13, which was proven by enzymatic tests that indicated higher β-glucosidase activity [90]. *T. hamatum* MHT1134 was applied to pepper cropping fields for 1 and 2 years that had been continuously planted for 1, 5, and 9 years. The application of MHT1134 increased the growth of certain microbes in the field, such as *Trichoderma* spp., *Chaetomium,* and *Actinobacteria*; hence, due to this abundance, the soil quality was significantly increased, and pathogenic microbes, such as *Fusarium* and *Gibberella*, decreased in abundance. *Fusarium* wilt disease was also significantly reduced in the pepper crops [91]. Whereas, recent studies described that, *T. hamatum* T21 genome assembly was obtained by CRISPR/Cas 9 system and Sg RNA efficient knock out method was successfully established in *T. hamatum* T21. This genome editing would be advantageous to investigate the mechanism of induced resistance and to know the secondary metabolite synthesis pathways in *T. hamatum*. Hence, genome editing of *T. hamatum* would way path in functional analysis of biocontrol genes and elucidates the molecular mechanism of filamentous fungi in agricultural applications [92]. Moreover, *T. hamatum* also possesses a quality that causes significant weight loss in intermediate and well-decayed wood compared to nondecayed wood [93]. 

Moreover, *T. hamatum* FBL 587 possesses significant tolerance to dichlorodiphenyltrichloroethane (DDT); whereas, when *T. hamatum* FBL 587 was evaluated for catabolic versatility against 95 carbon sources, FBL 587 utilized most of the carbon substrates, showing high metabolic versatility and ecological functionality of using carbon sources [94]. The whole-genome sequence of FBL 587 exposed to DDT was analyzed and approximately 1706 upregulated genes were observed when FBL 587 was exposed to DDT. Moreover, the upregulation of metabolizing enzymes such as P450s and the downregulation of certain DDT-transforming enzymes such as epoxide hydrolases, flavin-dependent monooxygenases, and glycosyl and glutathione transferases were observed (Table 3). However, the exact metabolic pathway and the degrading enzymes that are responsible for DDT degradation need to be identified further [95]. *T. hamatum* was tested to analyze its potential to degrade polyvinyl chloride (PVC); however, after 110 days of PVC treatment of *T. hamatum*, there was only a slight reduction in the mass, but PVC was not degraded [96]. 

*T. hamatum* Th-16 sustains salt resistance in wheat and mung bean plants; moreover, Th-16 increased the growth of the plant, the chlorophyll content and the resistance of the plant under extreme salinity. On the other hand, a reduction in the oxidative stress markers H_2_O_2_ and MDA and a simultaneous increase in the activity of antioxidant enzymes such as SOD and CAT activity compared to control plants was observed [97] (Table 3). 

**Table 3:** *Trichoderma hamatum* and its other beneficial activities.

S: No	Compounds/Enzymes/Secondary Metabolites/Genes of *T. hamatum*	Activity	Reference
1.	4,6- dihydroxy 5- methoxy-6a-methylcyclohexa [de] indano [7,6 –e] cyclopenta [c] 2H- pyrane-1,9-dione	Inhibits the enzyme 5′ hydroxyaverantin dehydrogenase essential for aflatoxin biosynthesis	[86]
2.	Hemicellulose enzyme α galactosidase, cellulose endo-1, 4, β glucanase	Polysaccharide degradation	[87]
3.	Endoglucanase	Saccharification	[88]
4.	42 kDa endo chitinase gene *Tam*-*ch*	Enhanced chitinase activity	[89]
5.	Β-glucosidase gene	Increased β- glucosidase activity	[90]
6.	Upregulation of enzymes P450s and down regulation of epoxide hydrolases flavin-dependent monooxygenases, glycosyl, and glutathione transferases	DDT degradation	[95]
7.	Superoxide dismutase and catalase increase and reduction in H_2_O_2_ and myoadenylate deaminase	Salt resistance	[97]

*T. hamatum* genes and several compounds, secondary metabolites produced represent several beneficial activities.

## 8. Conclusions

*T. hamatum* is a symbiotic beneficial fungus that accommodates in the rhizosphere and as endophytes in several plants. Its relationship with plants manifests through a protection of the plants against plant pathogens and in turn promotes their growth. Apart from these, *T. hamatum* produces polysaccharide-degrading enzymes, such as AGL and EG, and certain DDT-degrading enzymes. Moreover, it increases salt resistance in plants by increasing SOD and CAT enzymes. *T. hamatum* can be further considered a potential biocontrol microorganism, and the study of its extracellular and intracellular enzymes and secondary metabolites would provide a path for the identification of novel compounds that possess several biocontrol properties.

## 9. Future Prospects

*T. hamatum* is a beneficiary fungus with several biocontrol activities; however, the related research published thus far has focused on its biocontrol activities, such as its antifungal, antibacterial, antiviral, insecticidal/pesticidal, and herbicidal properties. The mechanisms related to these activities need to be investigated further. Moreover, *T. hamatum* exhibits antiviral activity against TMV, but further research is required on other plant viruses and their mechanisms of antiviral activity. Limited research was available on *T. hamatum* and its herbicidal activity and antioxidant activity; further research into these activities would likely lead to significant agricultural and medical developments. *T. hamatum* also possesses antimicrobial properties; hence, analysis of its activity against human pathogens would provide a path toward understanding the identification of novel antimicrobial agents. Moreover, there is also a need for further investigation into the opportunistic pathogenicity of *T. hamatum* towards humans.

## Figures and Tables

**Table 2:** *Trichoderma hamatum* potentiality in upland plant growth promotion.

S: No	Plant	Treatment of *T. hamatum*	Activity	Reference
1.	*Theobroma cacao*	Seedlings	Increase in drought tolerance, stomatal conductance, net photosynthesis, and green fluorescence emission	[78]
2.	*Pinus radiata*	Seedlings and roots	Rhizosphere competence, root penetration, shoot growth promotion, increase in dry root weight	[79,81]
3.	*Solanum lycopersicum*	Seedlings	Lateral root development	[80]
4.	*Brassica campestris* L. spp. *Chinensis* var. *utilis*	Biofertilizer to soil	Increase in germination rate, height, fresh weight, yield of flowering, soil enzymes urease, phosphatase, and catalase	[82]
5.	*Begonia X tuberhybrida*	Root tubers	Increase in chlorophyll production, blooming size of the flower, uptake of micronutrients zinc, iron, boron	[83]
6.	*Gladiolus hybridus*	5 weeks old cultivation	Increase in chlorophyll a + b content, elongation of inflorescence, number of flowers, uptake of macronutrients phosphorous, potassium, calcium and micronutrients zinc, iron, boron	[84]
7.	*Stanhopea tigrina*	In vitro plant	100% in vitro plant survival	[85]

*Trichoderma hamatum* influenced several factors and enhanced the upland plant growth in several plants by its treatment to seedlings, roots, or in vitro conditions.
